# The Secretome of Human Neonatal Mesenchymal Stem Cells Modulates Doxorubicin-Induced Cytotoxicity: Impact in Non-Tumor Cells

**DOI:** 10.3390/ijms222313072

**Published:** 2021-12-03

**Authors:** Ana S. Serras, Sérgio P. Camões, Bernardo Antunes, Vera M. Costa, Flávio Dionísio, Volkan Yazar, Rui Vitorino, Fernando Remião, Matilde Castro, Nuno G. Oliveira, Joana P. Miranda

**Affiliations:** 1 Research Institute for Medicines (iMed.ULisboa), Faculty of Pharmacy, Universidade de Lisboa, 1649-003 Lisbon, Portugal; ana.serras@edu.ulisboa.pt (A.S.S.); sergiocamoes@campus.ul.pt (S.P.C.); bernardoantunes_94@hotmail.com (B.A.); mcastro@ff.ul.pt (M.C.); ngoliveira@ff.ul.pt (N.G.O.); 2 Associate Laboratory i4HB—Institute for Health and Bioeconomy, Faculty of Pharmacy, University of Porto, 4050-313 Porto, Portugal; veramcosta@ff.up.pt (V.M.C.); fdionisio@ff.up.pt (F.D.); remiao@ff.up.pt (F.R.); 3 UCIBIO—Applied Molecular Biosciences Unit, Laboratory of Toxicology, Department of Biological Sciences, Faculty of Pharmacy, University of Porto, 4050-313 Porto, Portugal; 4 Institute for Cell Engineering, Johns Hopkins School of Medicine, Baltimore, MD 21205, USA; volkanyazar1984@gmail.com; 5 LAQV-REQUIMTE, Mass Spectrometry Center, Department of Chemistry, University of Aveiro, 3810-193 Aveiro, Portugal; rvitorino@ua.pt; 6 Cardiovascular R&D Center, Department of Surgery and Physiology, Faculty of Medicine, University of Porto, 4200-319 Oporto, Portugal; 7 iBiMED, Department of Medical Sciences, University of Aveiro, 3810-193 Aveiro, Portugal

**Keywords:** breast cancer, doxorubicin, cardiotoxicity, mesenchymal stem cells, secretome, 3D cultures

## Abstract

Doxorubicin (Dox) is one of the most widely used treatments for breast cancer, although limited by the well-documented cardiotoxicity and other off-target effects. Mesenchymal stem cell (MSC) secretome has shown immunomodulatory and regenerative properties, further potentiated under 3D conditions. This work aimed to uncover the effect of the MSC-derived secretome from 3D (CM3D) or 2D (CM2D) cultures, in human malignant breast cells (MDA-MB-231), non-tumor breast epithelial cells (MCF10A) and differentiated AC16 cardiomyocytes, co-treated with Dox. A comprehensive proteomic analysis of CM3D/CM2D was also performed to unravel the underlying mechanism. CM3D/CM2D co-incubation with Dox revealed no significant differences in MDA-MB-231 viability when compared to Dox alone, whereas MCF10A and AC16 viability was consistently improved in Dox+CM3D-treated cells. Moreover, neither CM2D nor CM3D affected Dox anti-migratory and anti-invasive effects in MDA-MB-231. Notably, Ge-LC-MS/MS proteomic analysis revealed that CM3D displayed protective features that might be linked to the regulation of cell proliferation (CAPN1, CST1, LAMC2, RANBP3), migration (CCN3, MMP8, PDCD5), invasion (TIMP1/2), oxidative stress (COX6B1, AIFM1, CD9, GSR) and inflammation (CCN3, ANXA5, CDH13, GDF15). Overall, CM3D decreased Dox-induced cytotoxicity in non-tumor cells, without compromising Dox chemotherapeutic profile in malignant cells, suggesting its potential use as a chemotherapy adjuvant to reduce off-target side effects.

## 1. Introduction

Breast cancer is the most frequently diagnosed cancer and the second-leading cause of cancer death in women [1]. The use of anthracyclines, specifically doxorubicin (Dox) and epirubicin, is established as the first line of treatment in solid tumors such as breast cancer [2], as well as in adjuvant treatment. Dox, in particular, acts mainly by inducing DNA damage, inhibiting cancer cell proliferation, inducing cell cycle arrest and apoptosis. Although the exact mechanisms involved are not fully understood, it is well-established that Dox inhibits topoisomerase IIα, intercalates in DNA strands and stimulates the generation of reactive oxygen species (ROS) [3,4,5].

Despite its tumor-killing effect, Dox is also associated with several dose-related side effects in off-target non-tumor cells of which cardiotoxicity stands out [6]. Unquestionably, Dox-induced cardiotoxicity greatly impacts its clinical use, affecting the quality of life of patients and often leading to life-threatening conditions [6,7]. The mechanisms of Dox-mediated cardiomyopathy are multifactorial and not completely established. Despite the fact that inhibition of topoisomerase IIβ has been recently described in the heart [3,5], the most studied mechanisms are oxidative stress and dysfunction of mitochondrial bioenergetics (also via topoisomerase IIβ) [3,7,8,9,10,11]. Dox largely accumulates on cardiac tissue because of its affinity to cardiolipin and a higher sensitivity to oxidative insult [7]. As such, myocardial tissues are more susceptible to the effects of Dox. Moreover, unlike non-tumor cells, cancer cells are able to shift their metabolism and to mediate defensive mechanisms against ROS, through, e.g., preferential energy dependence on glycolysis, or by increasing multidrug resistance (MDR) transporters that exclude oxidative stress-generated by-products [12,13,14]. In this context, Dox displays different mechanisms in tumor and non-tumor cells, which may create opportunities to develop novel adjuvant strategies, aiming at decreasing off-target toxicity of Dox while not interfering with its chemotherapeutic activity.

Different approaches have explored mesenchymal stem/stromal cells (MSCs) for their unique immunomodulatory and anti-inflammatory properties, targeting a wide range of diseases, including cardiomyopathy [11,15,16,17,18]. MSCs have also been regarded as possible anticancer therapeutic agents since they exhibit an intrinsic ability to migrate towards tumors in which they secrete antineoplastic factors such as IFN-α/β [19]. Interestingly, some studies have reported a pro-tumorigenic role of MSCs [20,21,22,23,24], while others describe MSCs as tumor inhibiting agents [25,26,27,28,29,30,31], both in vitro and in vivo. Although yet unclear, some aspects, including MSC tissue of origin, cancer type and experimental methodologies adopted (i.e., the use MSCs per se vs. their secretome) have been suggested as playing a key role in the distinct outcomes reported. Indeed, studies involving umbilical cord (UC)-derived MSCs have usually shown to inhibit cancer growth as opposed to bone marrow (BM)- or adipose tissue (AT)-derived MSCs [23,24,28,29,30,31,32]. In addition, when exposed to a cancer-like environment, MSCs may differentiate into tumor supporting cells, such as tumor-associated fibroblasts (TAFs) [32], raising concerns about the use of these cells per se. Importantly, an overall anti-tumor effect of MSCs is often observed when its secretome/conditioned media (CM) is administered to tumor cells, thus pending the balance towards the development of novel cell-free based therapies [29,31].

The secretome of MSCs has previously been shown to attenuate cardiac remodeling and preserve cardiac function in a murine myocardial infarction model by reducing cardiomyocyte apoptosis, promoting capillary-like structure formation by endothelial cells and stimulating resident cardiac progenitor cell proliferation and activation [17]. Moreover, it has been reported that the modulation of the MSC culture conditions by resorting to three-dimensional (3D) systems allows the production of a secretome with enhanced therapeutic properties [16,18,33]. In sum, innovative approaches are clearly needed to shed light on the usefulness of CM from both 2D and 3D MSC cultures in the context of Dox–induced effects in both cancer and normal-type cells. In this context, the present study aimed for the first time to uncover the role and to evaluate the effect of the secretome of MSCs in human malignant breast cells and non-tumor cells (i.e., normal breast epithelial cells and cardiomyocytes) upon co-treatment with Dox, further unraveling the putative underlying mechanisms, through a comprehensive mechanistic proteomic analysis.

## 2. Results

### 2.1. MSC Conditioned Media Encloses Proteins Involved in Cytoprotection

**Figure 1:**
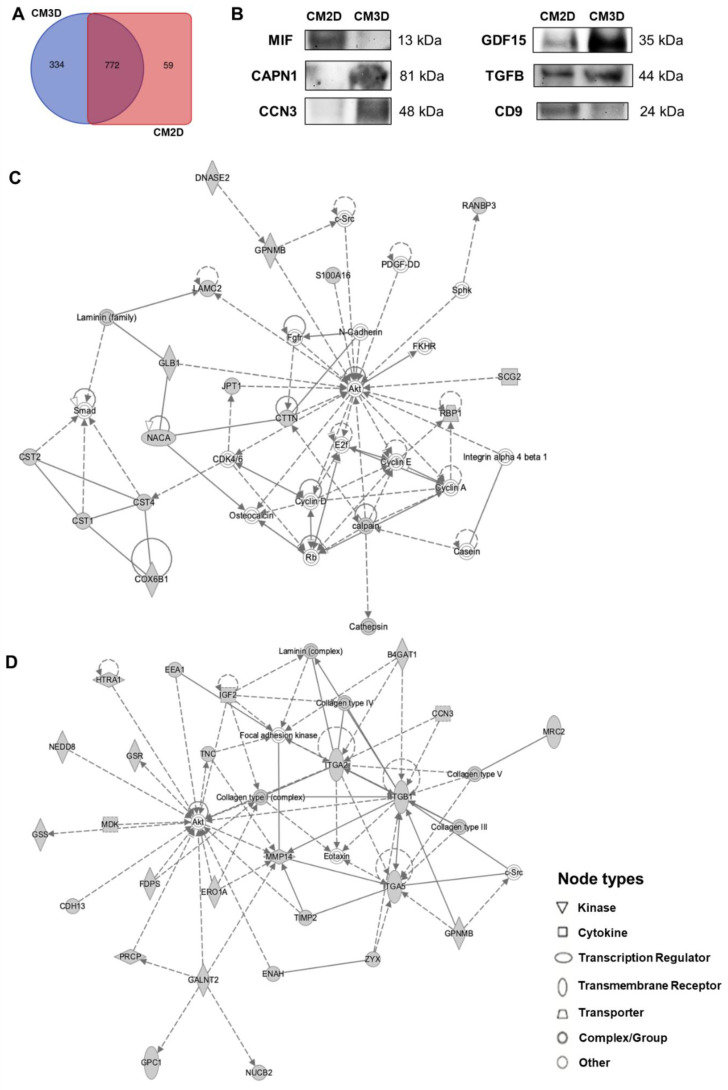
**Proteomic profiling of MSC CM revealed the presence of proteins involved in cytoprotection.** (**A**) Venn diagram shows the unique and shared protein numbers identified in CM3D and CM2D. (**B**) Confirmation of selected proteomic data by Western blot analysis of CAPN1, CCN3, MIF, GDF15, TGFB and CD9. (**C**) Network analysis of protein–protein interactions within CM3D with functions associated with cancer, carbohydrate metabolism and cardiovascular disease performed using the IPA software. (**D**) Network analysis of protein–protein interactions within CM3D unique and shared proteins with functions associated with the cardiovascular system development and function, cellular movement and tissue development performed using the IPA software. CM2D, conditioned medium derived from 2D cultures; CM3D, conditioned medium derived from 3D cultures; IPA, Ingenuity Pathway Analysis.

Recent knowledge supports that the mechanism of action of MSCs is mostly due to its paracrine effect rather than the cells per se. As such, in order to understand if the protein content of MSC CM (CM2D and CM3D) could suggest a role and justify or support its use as an adjuvant in breast cancer treatment, a Ge-LC-MS/MS proteomics followed by an Ingenuity Pathway Analysis (IPA) of both CM2D and CM3D was performed. As a result, a total of 1165 distinct proteins were identified, 1106 proteins within CM3D and 831 proteins within CM2D, showing also that both CM encompassed specific sets of proteins. Specifically, 772 proteins were found in CM3D and CM2D, whereas 334 proteins were identified as unique to CM3D and only 59 proteins unique to CM2D (Figure 1A).

The proteomic data analyzed by IPA allowed the identification of several proteins within the MSC secretome (CM2D and CM3D) involved in important biological processes. Specifically, a deeper analysis was focused on essential processes involved in cytoprotection and tumor development, e.g., cell proliferation, migration and invasion, inflammation and oxidative stress. Both CM presented proteins involved in proliferation of both tumor and non-tumor cells, of which CAPN1, CST1, LAMC2 and RANBP3 stood out in CM3D and GPC6, ILK, MAPK1, MIF and PICALM in CM2D. In addition, both CM presented a remarkable quantity of different proteins that have been associated with antioxidant and/or anti-inflammatory effects and may thus mediate the viability of non-tumor cells, such as AIFM1, ANXA5, CD9, CDH13, GDF15, GSR and TIMP2. Additionally, comparing both CM, a higher proportion of proteins with these properties were identified to be unique to CM3D, such as COX6B1, CCN3 and S100A16, whereas only MIF and SDC4 were unique to CM2D. Regarding cell migration, cytokines such as TGFB and IL6 are common to both CM, whilst proteins such as CCN3, MMP8, PDCD5 have been identified to be unique to CM3D and associated with decreased cell migration. In addition, GPC6, ILK, MAPK1, MIF and SDC4 were unique to CM2D, being linked to increased cell migration. Lastly, cell invasion is known to be regulated by proteins that were identified in both CM such as TIMP1 and TIMP2. Among those proteins, CAPN1 (cell proliferation) and CCN3 (inflammation and cell migration), exclusive for 3D samples; MIF (cell proliferation, migration and invasion), exclusive for 2D samples and GDF15 (inflammation), TGFB (cell proliferation) and CD9 (antioxidant and/or anti-inflammatory effects) for both samples were chosen for validation of the proteomic data by Western blot analysis (Figure 1B). 

IPA analysis of the secretome of MSCs also established protein–protein interaction networks. CM3D unique proteins formed a network related to cancer, carbohydrate metabolism and cardiovascular disease (Figure 1C), whereas mainly CM3D and CM2D shared proteins comprising a network related to the cardiovascular system development and function, cellular movement and tissue development (Figure 1D). Both networks are intimately associated with cell growth and survival and consistently suggest AKT, a serine/threonine-protein kinase, as a key player for the functional protein interactions involved in these biological processes. AKT kinase is involved in the regulation of various signaling downstream pathways including metabolism, cell proliferation, survival, growth and angiogenesis. The AKT kinase pathway stands among the most important components of the cell proliferation mechanism. 

### 2.2. The Effect of Doxorubicin in the Viability, Migration and Invasion of MDA-MB-231 Cells Is Maintained When Co-Administered with MSC Secretome

In order to understand if MSC CM could indeed result in biological effects, the outcome of administering MSC CM concomitantly with Dox was evaluated in both tumor (MDA-MB-231 cells) and non-tumor (MCF10A and AC16) cells (*see following sections*). 

**Figure 2:**
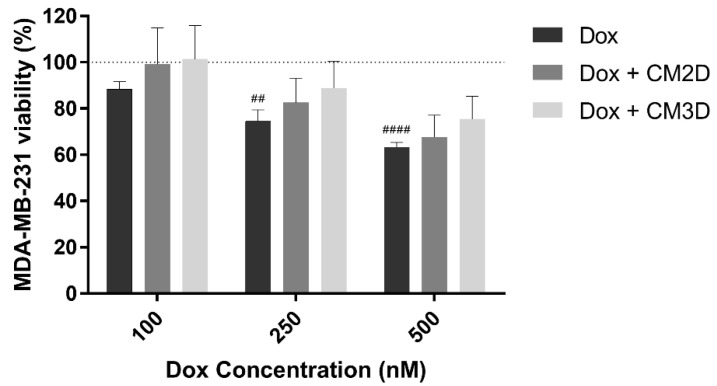
**Exposure of MDA-MB-231 cells to MSC CM did not significantly affect the cytotoxic effect of Dox.** MDA-MB-231 cells were incubated with 0–500 nM of Dox and both conditioned media, CM2D or CM3D, and cytotoxicity was evaluated by MTS reduction assay. Cell viability is expressed in percentage (mean ± SD, n = 3–6) to non-treated MDA-MB-231 cells (control). Grid line represents 100% cell viability (control). Statistical significance is expressed relative to control of non-treated cells, i.e., 100% viability as ^##^ *p* < 0.01, ^####^ *p* < 0.0001. CM2D, conditioned medium derived from 2D cultures; CM3D, conditioned medium derived from 3D cultures; Dox, doxorubicin.

The effect of concomitant exposure of CM3D or CM2D and Dox on the cell viability of the triple negative invasive breast cancer (TNBC) cell line MDA-MB-231 was assessed through a 48 h MTS assay. As expected, the exposure of human cancer cells to Dox led to a concentration-dependent cytotoxicity (Figure 2). Importantly, the same cytotoxic trend was observed upon combinatory exposure to CM2D or CM3D and Dox.

**Figure 3:**
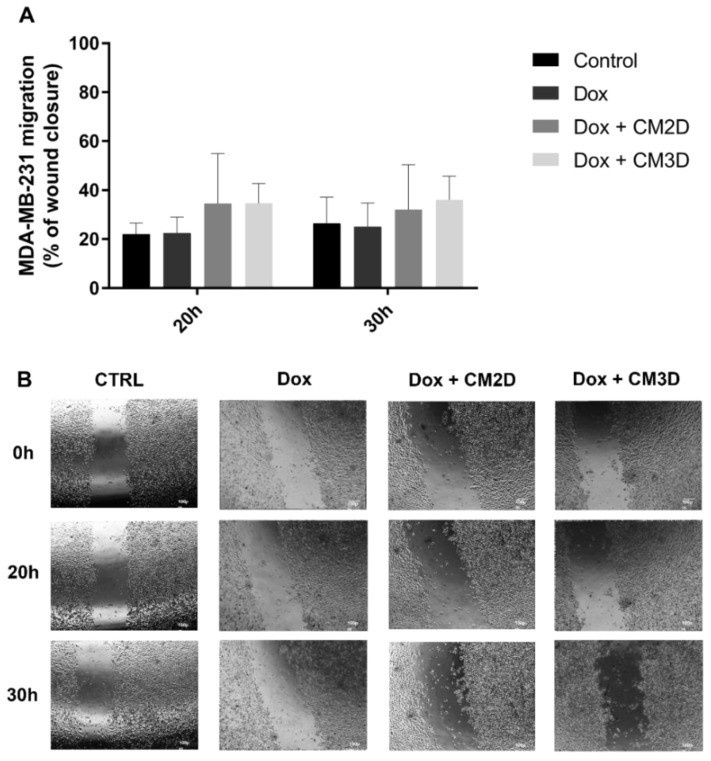
**Co-exposure of MDA-MB-231 cells to Dox and MSC CM did not affect cell migration.** (**A**) The effect of CM2D or CM3D in combination with Dox (100 nM) on the migration of MDA-MB-231 cells at the 20 and 30 h time points was evaluated by scratch assay. Cell migration is represented as percentage (mean ± SD, n = 3–6) of wound closure at the defined time points. (**B**) Representative images of scratch assays of MDA-MB-231 cells incubated with or without MSC CM (control) immediately after the scratches were made (0 h) and after 20 and 30 h. Magnification 4×, scale bar = 100 μm. CM2D, conditioned medium derived from 2D cultures; CM3D, conditioned medium derived from 3D cultures; Dox, doxorubicin.

**Figure 4:**
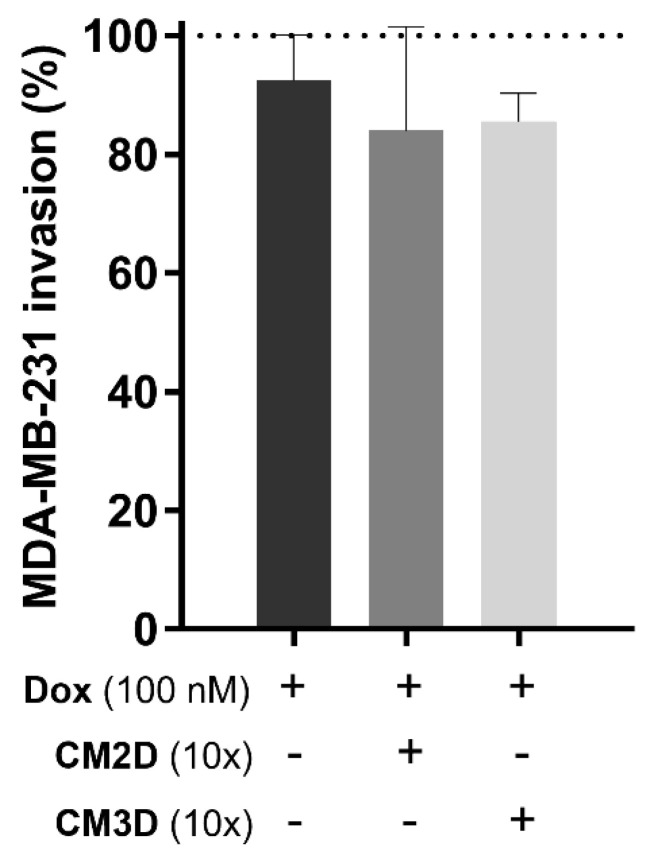
**Co-exposure of MDA-MB-231 cells to CM3D or CM2D maintained the anti-invasion effect of Dox.** The effect of CM2D or CM3D in combination with Dox (100 nM) on the chemoinvasion ability of MDA-MB-231 cells was evaluated by transwell assay. Results are expressed in percentage to control (mean ± SD, n = 4–6). Grid line represents 100% cell viability (control). CM2D, conditioned medium derived from 2D cultures; CM3D, conditioned medium derived from 3D cultures; Dox, doxorubicin.

The relative capacity of CM3D/CM2D in combination with Dox (100 nM) to influence MDA-MB-231 cell migration and invasion was also evaluated by in vitro scratch and transwell assays, respectively, and compared to Dox alone (Figure 3 and Figure 4). Importantly, under these conditions, a non-cytotoxic concentration of Dox (100 nM) was adopted in both assays in order to guarantee that the observed results are due to a migratory or invasive effect rather than cell viability. Figure 3B shows representative images of MDA-MB-231 cell migration at 0, 20 and 30 h after scratch where no statistical significance was observed between combinatory treatment of Dox and CM3D/CM2D and Dox alone (Figure 3A). Likewise, the co-administration of CM with Dox did not alter the effect of Dox alone in the ability of MDA-MB-231 cells to invade surrounding tissues (Figure 4). 

### 2.3. MSC Secretome Ameliorates the Cytotoxic Effect of Doxorubicin in Non-Tumor Breast Epithelial Cells

**Figure 5:**
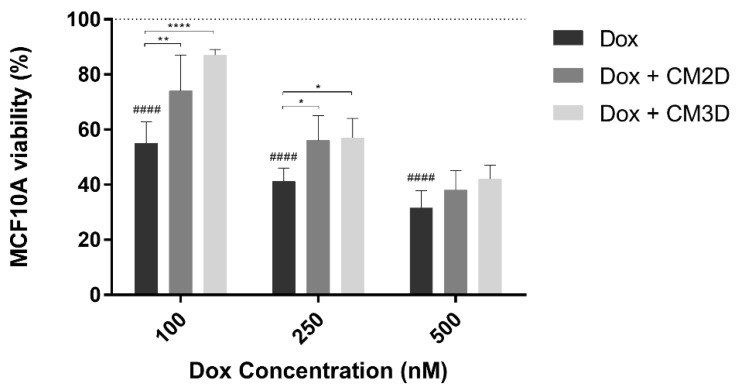
**Combinatory exposure of MCF10A cells to MSC CM increased the viability of non-tumor breast cells exposed to Dox.** The MCF10A cells were incubated with 0–500 nM of Dox alone and in combination with both conditioned media, CM2D or CM3D, and evaluated by MTS reduction assay. Cell viability is expressed in percentage (mean ± SD, n = 3–6) to non-treated MCF10A (control). Grid line represents 100% cell viability (control). Statistical significance is expressed relative to Dox as * *p* < 0.05, ** *p* < 0.01, **** *p* < 0.0001 and to control of non-treated cells, i.e., 100% viability as ^####^ *p* < 0.0001. CM2D, conditioned medium derived from 2D cultures; CM3D, conditioned medium derived from 3D cultures; Dox, doxorubicin.

To assess the effect of the secretome of MSCs on the cytotoxicity of Dox in non-tumor breast epithelial MCF10A cells, a MTS assay was used. Similar to tumor cells, Dox exposure led to a concentration-dependent effect on cell viability (Figure 5). Conversely, the concomitant exposure of MCF10A cells to Dox and CM3D resulted in an increase in the percentage of cell viability of approximately 32 ± 6% (*p* < 0.0001) and 16 ± 2% (*p* < 0.05) for Dox concentrations of 100 and 250 nM, respectively, when compared to cells treated with Dox. This beneficial effect was also observed, although to a lesser extent, within combinatory treatment of Dox and CM2D with an enhanced cell viability of 19 ± 5% (*p* < 0.01) and 15 ± 4% (*p* < 0.05), for 100 and 250 nM of Dox, respectively. 

### 2.4. The Cytotoxic Effect of Doxorubicin on Cardiomyocytes Is Ameliorated by the MSC Secretome from 3D Cultures

**Figure 6:**
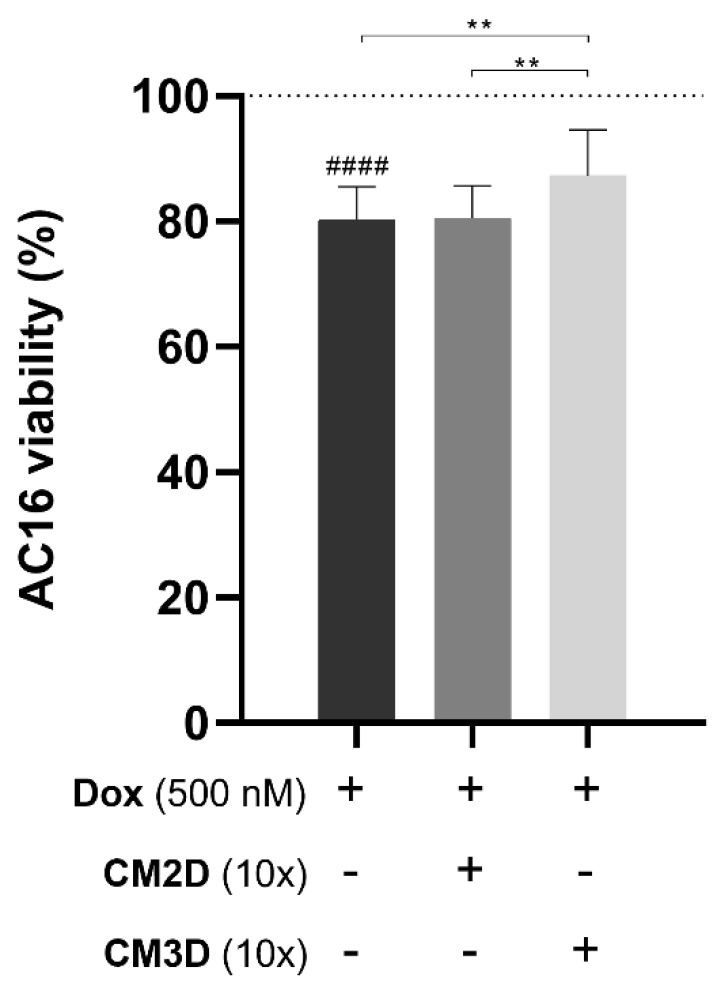
**Exposure of AC16 cardiomyocyte cells to CM3D increased the viability of cells exposed to Dox.** The differentiated AC16 cells were incubated with 500 nM of Dox alone or in combination with CM2D or CM3D being the cytotoxicity evaluated by MTS reduction assay. Cell viability is expressed in percentage (mean ± SD, n = 3–6) to non-treated differentiated AC16 cells (control). Grid line represents 100% cell viability (control). Statistical significance is expressed relative to Dox as ** *p* < 0.01 and to control of non-treated cells, i.e., 100% viability as ^####^ *p* < 0.0001. CM2D, conditioned medium derived from 2D cultures; CM3D, conditioned medium derived from 3D cultures; Dox, doxorubicin.

To evaluate the effect of the CM2D/CM3D on Dox-treated cardiomyocytes, AC16 differentiated cells were used to establish an in vitro cardiac model [34]. Dox exposure led to a decrease in cell viability of approximately 20 ± 5% (*p* < 0.0001; Figure 6). Moreover, the concomitant exposure to CM2D and Dox did not show significant improvement in cell survival (82 ± 7% Dox and CM2D vs. 80 ± 5% Dox alone; n.s.). On the other hand, the combinatory treatment of Dox and CM3D presented a significantly higher cell viability when compared to the group treated with Dox alone (87 ± 7% Dox and CM3D, *p* < 0.01) or with Dox and CM2D (*p* < 0.01), emphasizing the beneficial effect of CM3D (Figure 6).

## 3. Discussion

The side effects linked to the chemotherapeutic treatment of breast cancer with doxorubicin remain a major clinical concern. The application of the secretome of MSCs has been investigated regarding its active role in immunomodulation and regeneration processes [15,16,17,18,35], outlining its great potential in several pathological conditions. However, its effects within cancer setting have not yet been determined. In this study, we aimed at understanding the impact of the secretome of MSCs in combination with clinically relevant doses of Dox in both tumor and non-tumor cells.

According to our findings, neither CM3D nor CM2D significantly interfered with Dox treatment regarding cancer cell viability, the first indicator of safety for applicability in these conditions. Hendijani et al. have also found that the CM of MSCs did not promote tumor cell growth or resistance to Dox in lung cancer cells [36]. Remarkably, herein, CM presented a cytoprotective effect towards Dox cytotoxicity more pronounced in non-tumor breast epithelial cells and cardiomyocytes than in breast cancer cells. Hence, we firstly hypothesize that MSC CM comprises pro-proliferative factors. Indeed, several pro-proliferation proteins were uniquely identified in CM2D such as PICALM, which positively regulate cell proliferation [37] and GPC6, MAPK1, ILK and MIF, all involved in cell proliferation, migration and invasion, including that of tumor cells [38,39,40,41,42], cardiomyocytes and endothelial cells [38,39]. Several proteins were also uniquely identified in CM3D, such as RANBP3, involved in cell proliferation by negative regulation of TGF-β signaling [43]; CAPN1, mediating cell apoptosis, survival and migration [44] and CST1 and LAMC2, both involved in positive cell proliferation, migration and invasion [45,46]. Interestingly, as shown in both IPA-generated networks, most of the proteins identified in this study have been reported to modulate or be modulated by AKT. Indeed, the AKT signaling pathway is involved in cell death and survival responses of both tumor and non-tumor cells by induction of pro-angiogenic factors and inhibition of autophagy and pro-apoptotic factors [47]. Thus, all these proteins highlight the potential role of the MSC CM as modulator of cell proliferation and survival. 

Reported differences in Dox mechanism of toxicity between non-tumor and tumor cells may be linked to distinct responses of cancer and non-tumor cells to oxidative stress [12]. Dox anticancer effects are mainly related to topoisomerase IIα inhibition rather than this pro-oxidant effect [3,5], whereas in non-tumor cells, topoisomerase IIβ inhibition and oxidative stress are considered the main mechanisms [3,7,8,9,10,11]. Zhang et al. [35] have reported that CM from induced pluripotent stem cell-derived MSCs (iMSCs) effectively decreased ROS formation in cardiac cells treated with Dox when compared to the drug alone, mainly through the secretion of MIF and GDF15. Thus, a second mechanism of the MSC secretome, besides stimulating cell proliferation, may be behind these distinct effects. In this study, MIF and GDF15 were identified in CM2D and in both MSC CM, respectively. Moreover, proteins found in both CM such as AIFM1, CD9 and GSR have been associated with the maintenance of the redox status [48,49,50], whilst ANXA5, CDH13, GDF15 and TIMP2 have shown not only antioxidant but also anti-inflammatory effects [35,51,52,53,54]. Notwithstanding, CM3D consistently showed better results when in combination with Dox compared to the effect of CM2D and Dox. When cultured in 3D conditions, MSCs displayed increased secretion of pro-angiogenic and anti-fibrotic factors, e.g., VEGF, FGF-2 and HGF, when compared to cells in traditional 2D cultures [16,18,33,55]. Furthermore, upon proteomic analysis of CM3D, a variety of proteins stood out, including CCN3, a pro-angiogenic, anti-inflammatory and anti-fibrotic protein [56,57], COX6B1 that has been shown to reduce cell apoptosis and ROS levels in non-tumor cells by regulation of mitochondrial function [58], and S100A16, involved in cardiomyocyte apoptosis and cardiac hypertrophy reduction [59]. Within CM2D, fewer proteins such as SDC4, which presents anti-apoptotic and anti-hypertrophic effects in the heart [60], and MIF were identified. All these MSC-secreted factors may have contributed to the enhanced viability of non-tumor cells, particularly in the case of CM3D. This adds strength to the assumption that the secretome obtained from MSCs in 3D cultures is more physiologically relevant. 

Along with cell proliferation, cell migration and invasion are also essential to understand the effect of CM when concomitantly added with Dox to tumor cells. Scratch assay results showed that MDA-MB-231 migration ability was slightly increased, although non-significant, upon treatment with Dox and CM2D or CM3D. IL-6 has been reported to be present in higher levels in CM2D than in CM3D [18] and has also been associated with the stimulation of the migration of breast cancer cells by induction of AKT, MAPK and STAT3 phosphorylation [61]. Moreover, proteins exclusively identified in CM2D such as GPC6, ILK, MAPK1, MIF and SDC4 have been associated with positive regulation of cell migration [38,39,40,42,62]. On the other hand, CCN3, PDCD5 and MMP8 were found to be uniquely identified in CM3D and have been associated with negative regulation of cell migration [63,64,65]. Metalloproteinases (MMPs) have been correlated with increased tumor cell migration and invasion through degradation of the basement membrane. However, it has been suggested that MMP-8 has a tumor suppressive role in breast cancer cells that, in contrast to gastric and liver tumors, leads to inhibition of migration, invasion and metastasis, both in vitro and in vivo [64]. This interesting role in breast cancer may be due to the inhibition of TGF-β with subsequent activation of programmed cell death 4 (PDCD4), decrease of MMP-3 and MMP-9 and increase of cell adhesion [64]. 

It is worth mentioning that cell migration and invasion are two distinct events within cancer progression and metastization [66]. An important anti-invasion mechanism of Dox has been reported to be the downregulation of MMPs [67]. Clarke et al. [29] found that the presence of TIMP-1 and TIMP-2, which are inhibitors of MMPs, in the secretome of immortalized BM-MSC led to the inhibition of breast cancer cell movement, with a better response towards invasiveness than migration. Indeed, our proteomic analysis identified TIMP1 and TIMP2 in both CM, which may thus exert a cumulative effect on the downregulation of MMPs induced by Dox. 

Overall, our results showed that the secretome of MSCs exhibited different effects in tumor and non-tumor cells. When combined with Dox, the MSC secretome did not significantly affect its cytotoxic, anti-migration or anti-invasive effects. Moreover, a partial but significant protection in cell viability was observed in both MCF10A and AC16 cells upon concomitant treatment, while the Dox-related cytotoxic effect was maintained in MDA-MB-231 cells. Importantly, CM3D consistently showed better results than CM2D, and several proteins were identified which may be related to these differential effects. These results may lead the way for the use of the secretome of MSCs, specifically from 3D cultures, to reduce the undesirable side effects of Dox without compromising its anticancer activity.

## 4. Materials and Methods

### 4.1. Reagents

Minimum essential medium Eagle alpha modification (α-MEM), Dulbecco’s modified Eagle’s medium (DMEM), Ham’s F-12 nutrient mixture medium (F12), penicillin–streptomycin solution, insulin solution from bovine pancreas, hydrocortisone, cholera toxin, human epidermal growth factor, gelatin, fibronectin and Dox were obtained from Sigma-Aldrich (St. Louis, MO, USA). Horse and fetal bovine sera (FBS) and trypsin/ethylenediamine tetraacetic acid (EDTA) solution were obtained from Gibco^®^ (Thermo Fisher Scientific, Waltham, MA, USA). 3-(4,5-dimethylthiazol-2-yl)-5(3-carboxymethonyphenol)-2-(4-sulfophenyl)-2H-tetrazolium (MTS) was purchased from Promega (Madison, WI, USA). An 800 µM stock solution of doxorubicin hydrochloride (Dox) was prepared in H_2_O MilliQ, aliquoted and stored at −20 °C until further use.

### 4.2. MSC Isolation

This study was approved by the Ethics Committee of the Hospital Dr. José de Almeida (Cascais, Portugal), in the scope of a research protocol between ECBio (Research & Development in Biotechnology, S.A.) and HPP Saúde (Parcerias Cascais, S.A.). Umbilical cord donations, with written informed consent, as well as umbilical cord procurement were carried out according to Directive 2004/23/EC of the European Parliament and of the Council of 31 March 2004 on setting standards of quality and safety for the donation, procurements, testing, processing, preservation, storage and distribution of human tissues and cells. MSCs are a population of umbilical cord tissue-derived human neonatal mesenchymal stromal cells and were isolated as described in the patent WO/2009/044379, developed by ECBio, S.A. [68] from umbilical cords of healthy new-born babies, upon informed consent of healthy parturients, as previously described [69]. Cells were cryopreserved in α-MEM containing 10% dimethyl sulfoxide (DMSO) stock solution and 20% FBS, using a controlled rate of temperature decrease. When needed, MSCs cryopreserved between passage (P)3 and P5 were thawed and further expanded. MSCs keep their phenotype until at least 55 cPDs (P22) before reaching senescence [16].

### 4.3. Conditioned Media Production from MSC Cultures

The production of conditioned media from 2D cultures (CM2D) and 3D cultures (CM3D) was performed according to a previously optimized protocol [16,18]. Both types of CM were produced from cells having undergone the equivalent number of cPDs; equivalent cell/volume ratio, i.e., the volume for CM3D production was adjusted to obtain a conditioning volume per cell equivalent to that in the 2D system and the same conditioning time (48 h). For the production of CM2D, cells were seeded at an inoculum of 1 × 10^4^ cells/cm^2^ in 175 cm^2^ t-flasks and maintained in medium supplemented with 5% FBS until reaching 90% confluence, generally at day 3. After carefully washing the cells, medium was replaced by α-MEM without FBS, at a final volume of 25 mL. After a 48 h conditioning period, CM2D was collected under sterile conditions. CM3D was obtained through the cell inoculation and expansion according to [16,18]. After 24 h, FBS concentration was reduced to 5%, and the cells were maintained in these conditions for 3 days. At day 5, medium was replaced by α-MEM without FBS. After a 48 h conditioning period, CM3D was then collected under sterile conditions. CM3D and CM2D were used at a final concentration of 10×, achieved by using 3 kDa cut-off centrifugal concentrators (Millipore^®^, Merck, Darmstadt, Germany) as per manufacturer’s recommendations. The control consisted of MSC medium, which was never in contact with cells. All samples were stored aseptically at −80 °C until further use.

### 4.4. LC-MS/MS Analysis

A total of 30 μg of each sample was isolated, and the volume was adjusted to 300 μL with 4% SDS in 0.5 M Tris pH 6.8. A total of 6 samples were prepared for LC-MS/MS analysis with the S-Trap^®^ Micro Spin Column (Protifi, Farmingdale, NY, USA) digestion protocol according to the manufacturer’s instructions with slight modifications. Briefly, all samples were centrifuged for 8 min at 13,000× *g*, and proteins in the supernatant were first reduced by addition of 20 mM DTT and incubation for 10 min at 95 °C and then alkylated by addition of 40 mM iodoacetamide and incubation for 30 min at RT in the dark. Proteins were digested with trypsin (Promega; 1/25, *w/w*) by adding trypsin in 50 mM TEAB solution to the micro column for 1 h at 47 °C. Peptides were eluted according to the manufacturer’s instructions.

Purified peptides were dried and re-dissolved in solvent A (0.1% trifluoroacetic acid (TFA) in water/acetonitrile (ACN;98:2, *v/v*)), and approximately 2 μg of each sample was injected for LC-MS/MS analysis on an Ultimate 3000 RSLC nanoLC (Thermo Fisher Scientific) in-line connected to an LTQ-Orbitrap Elite (Thermo Fisher Scientific). Trapping was performed at 10 μL/min for 4 min in solvent A on a 20 mm trapping column (made in-house, 100 μm internal diameter, 5 μm beads, C18 Reprosil-HD, Dr. Maisch, Germany), and the sample was loaded on a 200 cm long micro pillar array column (PharmaFluidics, Ghent, Belgium) with C18-endcapped functionality mounted in the Ultimate 3000′s column oven at 50 °C. For proper ionization, a fused silica PicoTip emitter (10 μm inner diameter; New Objective, Littleton, MA, USA) was connected to the μPAC^®^ outlet union, and a grounded connection was provided to this union. Peptides were eluted by a non-linear increase from 1 to 55% MS solvent B [0.1% formic acid (FA) in water/ACN (2:8, *v/v*)] over 115 min, first at a flow rate of 750 then at 300 nL/min, followed by a 15-min wash reaching 99% MS solvent B and re-equilibration with MS solvent A (0.1% FA in water). 

The mass spectrometer was operated in data-dependent, positive ionization mode, automatically switching between MS and MS/MS acquisition for the 20 most abundant peaks in a given MS spectrum. The source voltage was set to 2.7 kV, and the capillary temperature was 275 °C. In the LTQ-Orbitrap Elite, full scan MS spectra were acquired in the Orbitrap (*m/z* 300−2000, automatic gain control) with a resolution of 60,000 (at 400 *m/z*). The 20 most intense ions fulfilling predefined selection criteria were then isolated in the linear ion trap and fragmented in the high-pressure cell of the ion trap.

### 4.5. MS Data Analysis

The mass spectrometry proteomics data have been deposited in the ProteomeXchange Consortium via the PRIDE [70] partner repository with the dataset identifier PXD029795. Data analysis was performed with MaxQuant (version 1.6.8.0) using the Andromeda search engine with default search settings including a false discovery rate set at 1% on the PSM, peptide and protein level. Spectra were searched against the human protein sequences in the Uniprot database (database release version of June 2018), containing 20,960 sequences (www.uniprot.org, accessed on 16 December 2020). The mass tolerance for precursor and fragment ions was set to 4.5 and 20 ppm, respectively, and enzyme specificity was set as C-terminal to arginine and lysine, with a maximum of two missed cleavages. Variable modifications were set to oxidation of methionine residues and acetylation of protein N-termini, while carbamidomethylation of cysteine residues was set as a fixed modification. 

### 4.6. Ingenuity Pathway Analysis (IPA)

Gene symbols of the significant proteins identified were uploaded to the Ingenuity Pathway Analysis (IPA v10.2020, QIAGEN, Hilden, Germany) server for in-depth knowledge analysis using the “Core Analysis” function (Fisher’s exact test (FET) *p*-value: 1 × 10^−3^). The upstream regulators were predicted by IPA using the default settings. The Venn diagram was generated using the “venn()” function in the R package gplots v3.0.1.1. The gene list enrichment analysis platform, EnrichR v01.07.2020 [71], at https://amp.pharm.mssm.edu/Enrichr/ (accessed on 16 December 2021) was used with the following libraries in this study: DisGenNet RDF v7.0, Broad’s Project Achilles, GO (v2015), Jensen Disease and Compartments (v2020), ReactomePA (v2015), WikiPathways (2019) and Pfam Domains (v2019). STRING v11 protein association network analysis was performed with a minimal interaction score of 0.400 (FET *p*-value: 1 × 10^−3^) [72].

### 4.7. In Vitro Cell Viability Assay

The effect of CM2D and CM3D in cell viability, either alone or in combination with Dox, was evaluated in the two human mammary cell lines and in the differentiated human cardiomyocytes using the MTS reduction assay.

MDA-MB-231 and MCF10A were acquired from ATCC (Manassas, VA, USA) and seeded in 96-well plates at a density of 6.5 × 10^3^ cells/cm^2^ and 4.0 × 10^3^ cells/cm^2^ per well, respectively, and kept in a humidified atmosphere at 37°C and 5% CO_2_. MDA-MB-231 were cultured in DMEM supplemented with 10% FBS and MCF10A in DMEM/F12 medium, containing 5% horse serum, 100 U/mL penicillin, 100 μg/mL streptomycin, 0.01 mg/mL insulin, 0.5 µg/mL hydrocortisone, 0.1 µg/mL cholera toxin and 20 ng/mL human epidermal growth factor. After 48 h of plating, cells were exposed to CM2D/CM3D (10× concentrated) alone or in combination with Dox (100 to 500 nM) for 48 h. Cells were also incubated with α-MEM and H_2_O MilliQ (solvents for CM and Dox, respectively) in complete cell culture medium as controls. Afterwards, medium was discarded, 100 µL of cell culture medium with 20 µL of MTS was added and cells were incubated for 1 h at 37 °C, absorbance was measured at 490 nm using a microplate spectrophotometer (SPECTROstar Omega, BMG LABTECH, Offenburg, Germany). Three independent experiments in triplicates were performed. Results were expressed as percentage relative to control, which was considered as 100% cell viability.

AC16 human cardiomyocyte cells were used until the 10^th^ passage as recommended [34] and were seeded at a density of 32.5 × 10^4^ cells/cm^2^ in gelatin- and fibronectin-coated 48-well plates (0.02% and 0.0005% *w/v*). AC16 cells were cultured in DMEM/F12 medium supplemented with 12.5% FBS, 100 U/mL penicillin and 100 μg/mL streptomycin and kept at 37 °C in a humidified atmosphere with 5% CO_2_ during the proliferative stage. After 24 h, medium was changed to DMEM/F12 supplemented with 2% horse serum, 100 U/mL penicillin and 100 μg/mL streptomycin. After 24 h of differentiation, AC16 cells were incubated with Dox (100 to 500 nM) and/or CM2D/CM3D (10× concentrated) for 24 h. The medium was removed, and fresh medium was added with MTS (final concentration on the well of 158.5 μg/mL). Similarly, the cells were maintained at 37 °C and 5% CO_2_ for 1 h, and the absorbance at 490 nm was measured. Three to six independent experiments were performed, each in quadruplicate. Results were expressed as percentage relative to control, which was considered as 100% cell viability.

### 4.8. Immunoblotting Analysis

A total of 30 μg of total protein from each condition was resolved by SDS-PAGE in 12% polyacrylamide gels prepared as described by [73]. Gels were blotted onto PVDF membranes, which were incubated with primary antibody diluted in 5% BSA (anti-CAPN1 diluted 1:500 (#2556; Cell Signaling, Danvers, MA, USA), anti-TGFB diluted 1:100 (ab92486; Abcam, Cambridge, UK), anti-CCN3, anti-GDF15 and anti-MIF diluted 1:100 and anti-CD9 diluted 1:200 (sc-136967, sc-377195, sc-271631, sc-13118 respectively; Santa Cruz Biotechnology, Heidelberg, Germany) overnight at 4 °C, washed and incubated with horseradish peroxidase-conjugated anti-mouse (R&D Systems, Minneapolis, MN, USA) or anti-rabbit (Jackson ImmunoResearch, Cambridgeshire, UK) antibodies for 2 h at RT. Immunoreactive bands were detected by enhanced chemiluminescence ECL (Millipore^®^) according to the manufacturer’s instructions, and images were recorded using a ChemiDoc XRS System (Bio-Rad Laboratories, Hercules, CA, USA). Protein loading control was performed with Ponceau S staining.

### 4.9. In Vitro Scratch Assay

Breast cancer cell migration was assessed under exposure to CM2D/CM3D (10× concentrated) and/or Dox (100 nM) through an in vitro scratch assay, performed in accordance with [16,18]. Briefly, 2 × 10^5^ MDA-MB-231 cells were seeded in 24-well plates in complete culture medium. After 24 h, a scratch was performed using a 200 µL sterile pipette tip, and cells were incubated in serum-free medium containing the test compounds. Scratches were evaluated microscopically (Motic AE 2000 inverted microscope, Barcelona, Spain), and three images of each scratch were recorded using a Moticam 2500 at defined time points: 0, 20 and 30 h. Cell migration was measured in Motic Images PLUS v2.0 software by calculating scratch closure, given as the total migrated area after treatments in relation to the initial scratch area at 0 h (considered as 0% wound closure).

### 4.10. In Vitro Chemoinvasion Assay

The chemotactic invasion of MDA-MB-231 cells was evaluated in a transwell assay as previously described [67]. The assay was performed in 24-well plates containing transwell inserts with transparent PET membranes of 8 µm pores (Corning, Corning, NY, USA) overlaid with Matrigel (Corning) diluted in serum-free medium (1:30). Briefly, 1 × 10^5^ cells were seeded in the upper chamber in serum-free medium, while complete culture medium (chemoattractant) was added to the lower chamber. The test compounds were added to both chambers, and cells were incubated for 24 h at 37 °C in a 5% CO2 in air atmosphere. Cells in the upper part of the insert were removed using a cotton swab, and the invading cells in the lower part of the inserts were fixed with 96% cold ethanol for 15 min at 4 °C, stained with a 0.1% crystal violet solution for 10 min and then left to dry at 4 °C. The amount of cell attached dye was dissolved with a 1% acid acetic in ethanol solution, and optic density was measured at 595 nm using a microplate spectrophotometer (SPECTROstar Omega, BMG LABTECH). Results were expressed as percentage relative to control.

### 4.11. Statistical Analysis

All statistical analyses of cell data were performed in GraphPad Prism software (La Jolla, CA, USA). Comparisons were analyzed by one-way and two-way ANOVA followed by Tukey’s post hoc test. Results are expressed as mean ± standard deviation (SD), and *p*-values are presented for statistically significant results (* *p* < 0.05, ** *p* < 0.01, *** *p* < 0.001 and **** *p* < 0.0001).

## 5. Conclusions

In this paper, we present an approach for using the secretome of MSCs in the perspective of adjuvant treatment to Dox chemotherapy for breast cancer. Remarkably, we demonstrated that the MSC secretome, mainly CM3D, was significantly effective in protecting non-tumorigenic mammary epithelial cells and cardiomyocytes from the cytotoxic effect of Dox at a clinically relevant concentration, while it did not affect breast cancer cells. Moreover, we showed that the protective role of MSC secretome on non-tumor cells was greater in the form of CM3D, along with less interference with Dox. We further proposed that the mechanism behind CM3D effects may be due to the presence of proteins involved in biological processes such as cytoprotection, namely by regulating cell proliferation (CAPN1, CST1, LAMC2, RANBP3), migration (MMP8, PDCD5) and invasion (TIMP1, TIMP2), oxidative stress (COX6B1, AIFM1, CD9, GSR) and inflammation (CCN3, ANXA5, CDH13, GDF15). This work contributed to the development of novel adjuvant anticancer therapies, envisioning safer and more efficient use of chemotherapeutic agents.

## Data Availability

The mass spectrometry proteomics data have been deposited in the ProteomeXchange Consortium via the PRIDE partner repository with the dataset identifier PXD029795.

## Abbreviations

AT	adipose tissue
BM	bone marrow
CM	conditioned medium (secretome)
CM2D	secretome/conditioned medium from 2D cultures of mesenchymal stem cells
CM3D	secretome/conditioned medium from 3D cultures of mesenchymal stem cells
Dox	doxorubicin
iMSCs	induced pluripotent stem cell-derived MSCs
IPA	ingenuity pathway analysis
MDR	multidrug resistance
MSCs	mesenchymal stem/stromal cells
MTS	3-(4,5-dimethylthiazol-2-yl)-5-(3-carboxymethoxyphenyl)-2-(4-sulfophenyl)-2H-tetrazolium
ROS	reactive oxidative species
TAFs	tumor-associated fibroblasts
TNBC	triple negative invasive breast cancer
UC	umbilical cord
